# An Unusual Case of Perirenal Abscess Caused by *Campylobacter* and *Peptostreptococcus* Species

**DOI:** 10.1155/2022/4028085

**Published:** 2022-01-13

**Authors:** Krystal Hasel, Ahlaa Salim, Paul Adjei, Jeremy D. Gradon

**Affiliations:** Sinai Hospital, Baltimore, MD, USA

## Abstract

Intra-abdominal abscesses mostly derive from the intra-abdominal viscera. *Campylobacter* spp. are Gram-negative rods which are known to cause oral infections but rarely have been documented to cause extra-intestinal infections resulting in abscesses. We report an atypical case of *Campylobacter rectus* (*C. rectus*) and *Peptostreptococcus* spp. bacteria isolated from a perinephric abscess presenting as abdominal pain. Abscesses originating from outside the gastrointestinal tract have been reported in other similar case reports infecting the head, brain, and thoracic wall amongst others. The potential source and development of such a *Campylobacte*r infection could be due to multiple hypotheses. This is a first case report of perinephric abscess development. Studies have suggested person-to-person (fecal-oral) transmission along with insects serving as primary reservoirs. Seeding of bacteria through infections in the oral cavity or through infections in the bowel microperforations has also been considered as plausible reasons. Since *C. rectus* has been isolated in such rare instances, it should be kept in mind when considering differential diagnosis of potential causative agents for extra-oral infections such as invasive abscess formations.

## 1. Introduction

Intra-abdominal abscesses mostly derive from the intra-abdominal viscera. Gut flora including members of the *Enterobacteriaceae* family, viridans streptococcus group, and *Enterococcus* spp. and anaerobes such as *Bacteroides* spp. and *Candida* spp. have been frequently isolated from such cultures [1]. *Campylobacter* species are Gram-negative, spiral-shaped rods that usually colonize the oral cavity [2, 3]. Although diarrheal illnesses are known to be caused by *Campylobacter* spp., extra-intestinal infections resulting in abscesses have been observed and/or reported only in rare instances [2].

We report a case of perinephric abscess caused by *Campylobacter rectus* (*C. rectus*) and *Peptostreptococcus* spp. and review potential sources of this organism based on previous literature.

## 2. Case Report

**Figure 1:**
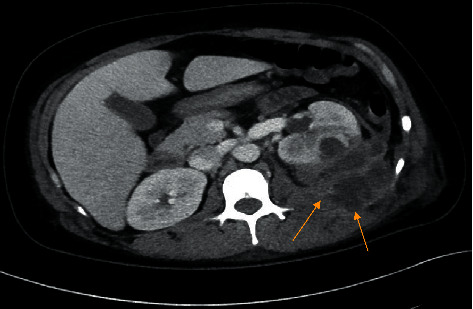
Computed tomography (CT) scan of the abdomen and pelvis showing left kidney and extensive expansion into the left retroperitoneum, psoas muscle, and possibly even the left posterior abdominal wall with multiple loculated abscesses.

A 40-year-old female with no significant past medical history presented with three-day history of left lower quadrant abdominal pain. The pain began in her left flank and subsequently radiated to her abdomen. It was associated with nausea, fever, and chills, and she denied having any diarrhea. She was febrile at 38.2°C. Laboratory findings showed leukocytosis of 16.9 K/MM3. Urinalysis was normal. Computed tomography (CT) scan of the abdomen and pelvis with intravenous (IV) contrast revealed an inflammatory process originating from the posterior left kidney with extensive expansion into the left retroperitoneum, psoas muscle, and possibly even the left posterior abdominal wall with multiple loculated abscesses (Figure 1). Piperacillin-tazobactam was empirically started for initial management. The patient underwent interventional radiology (IR) guided drainage on hospital day 2 (D2). A pigtail catheter was placed which drained purulent fluid, and culture revealed heavy growth of *Campylobacter rectus* and moderate growth of *Peptostreptococcus.* Antimicrobials were then changed to ampicillin-sulbactam and azithromycin. After six days of treatment, a repeat CT scan of the abdomen and pelvis was obtained which revealed a significant reduction in the size of the abscess. The multiloculated left perirenal abscess was now smaller in size but still extended into the left retroperitoneum and along the margin of the left psoas muscle. A colonoscopy was also performed which did not exhibit any intestinal abscess. After 10 days of hospitalization, the patient showed clinical improvement with resolution of flank pain, fever, and leukocytosis. The pigtail drain was removed on day 10. She was advised to take oral amoxicillin-clavulanate and azithromycin for 4 more days, completing a 14-day course of antibiotics. Unfortunately, the patient was lost to follow-up after discharge and was unable to participate in longitudinal care.

## 3. Discussion

The potential source and development of this patient's *Campylobacte*r infection remain unclear. A study in New Zealand suggested that person-to-person (fecal-oral) transmission was responsible for 4% of campylobacteriosis cases [3]. Unpasteurized dairy milk has also been found as an etiology of campylobacteriosis outbreaks [3]. In addition, other sources such as insects have also been proposed as reservoirs of *Campylobacter* species [3]. *C. rectus* has been isolated from the oral flora and is a well-known causative agent for periodontitis. In fact, a large-scale study from Finland found the presence of *C. rectus* in the saliva of 31.3% of healthy individuals [9].

*Peptostreptococcus* species are also anaerobic bacteria but are Gram-positive cocci. Although they are commonly found in the normal flora of the skin and gastrointestinal or genitourinary tract, these species have also been isolated as part of the normal flora of the oral cavity [10]. Perinephric abscesses can complicate urologic infections accounting for 75% of cases; however, they can also be due to hematogenous spread of infection to the kidney [11]. In our case, the patient's urinalysis and blood cultures were unremarkable, making primary urologic source very unlikely.

Risk factors associated with the development of extra-oral infections with *C. rectus* include poor dental hygiene, periodontal disease, dental abscess, and/or underlying comorbidities including diabetes, alcohol abuse, immunosuppression, and underlying malignancy [12]. Our patient was not known to have any of these listed risk factors and also denied any recent surgeries aside from a cesarean section several years prior.

The most likely source of the abscess here is a bowel microperforation. However, colonoscopy did not reveal any obvious bowel wall defect. Given the presence of both *Campylobacter* and *Peptostreptococcus* in the oral cavity, it is plausible that the patient developed the perinephric abscess via hematogenous spread from the oral cavity despite negative blood cultures.

A literature review in 2018 listed extra-oral infections caused by *C. rectus* which include abscesses in the brain, breast, palate, thoracic wall, and vertebra [12]. Reports of subdural empyema, sacroiliitis, necrotizing soft tissue infection of the thigh, septic cavernous sinus thrombosis, left acute otitis media, and mastoiditis have also been documented [12]. So far, there has been no reported case of perinephric abscess caused by *C. rectus.* We have listed a review of all reported abscesses associated with *C. rectus* in Table 1 [12].

## 4. Conclusion

We are reporting a case of a perinephric abscess caused by *Campylobacter rectus* and *Peptostreptococcus* species. *C. rectus* has been reported to cause extra-oral abscesses in various locations; however, this is the first case report of perinephric abscess development. Either undetected bowel microperforations or hematogenous spread from the oral cavity as in this case could be the potential pathogenesis behind extra-oral abscess formation, similarly hypothesized in other case reports. *C. rectus* should not be disregarded and underestimated as a potential causative agent for extra-oral infections such as invasive abscess formations.

## Figures and Tables

**Table 1:** Reported abscesses associated with *Campylobacter rectus.*

Author/year	Title	Number of patients for case series	Primary presentation	Comorbidities	Diagnosis	Treatment	Outcome
Spiegel and Telford/1984	Isolation of *Wolinella recta* and *Actinomyces viscosus* from an Actinomycotic Chest Wall Mass [4]	1	Cough with sputum	Chronic alcoholism	Thoracic wall abscess	Drainage, antibiotics	Recovery
Marrie and Kerr/1990	Brain Abscess due to *Wolinella recta* and *Streptococcus intermedius* [5]	1	Headaches, nausea, vomiting, anorexia, chills	n/a	Brain abscess	Drainage, antibiotics	Recovery
Han et al./2005	Oral *Campylobacter* Species Involved in Extraoral Abscess: A Report of Three Cases [6]	3	Fever, tenderness of the left breast	Lymphoma, neutropenia, nipple piercing	Breast abscess	Drainage, antibiotics	Recovery
De Vries et al./2008	*Campylobacter* Species Isolated from Extra-Oro-Intestinal Abscesses: A Report of Four Cases and Literature Review [2]	4	Lower back pain radiating to upper lower extremities	Chronic otitis, meningoradiculitis	Vertebral abscess	Drainage, antibiotics	Recovery
Mahlen and Clarridge/2008	Oral Abscess Caused by *Campylobacter rectus:* Case Report and Literature Review [7]	1	Dysphagia	Gastro-esophageal adenocarcinoma, smoker	Palate abscess	Drainage, antibiotics	Recovery
Martiny et al./2017	MALDI-TOF MS Contribution to the Diagnosis of *Campylobacter rectus* Multiple Skull Base and Brain Abscesses [8]	1	Vertigo, gait instability, repetitive falls	Dental extraction 10 weeks previously	Mastoiditis and brain abscess	Drainage, antibiotics	Recovery

## Data Availability

The data used to support the findings of this study are included within the article.
